# Detection of carbapenemase producing enterobacteria using an ion sensitive field effect transistor sensor

**DOI:** 10.1038/s41598-021-91202-6

**Published:** 2021-06-08

**Authors:** Stathis D. Kotsakis, Georgios Miliotis, Eva Tzelepi, Leonidas S. Tzouvelekis, Vivi Miriagou

**Affiliations:** 1 Laboratory of Bacteriology, Hellenic Pasteur Institute, Vas. Sofias 127, 115-21 Athens, Greece; 2 Microbiology Department, School of Medicine, National and Kapodistrian University of Athens, Mikras Asias 75, 115-26 Athens, Greece

**Keywords:** Bacteriology, Clinical microbiology, Infectious-disease diagnostics, Antimicrobials, Antimicrobial resistance

## Abstract

The timely and accurate detection of carbapenemase-producing *Enterobacterales* (CPE) is imperative to manage this worldwide problem in an effective fashion. Herein we addressed the question of whether the protons produced during imipenem hydrolysis could be detected using an ion sensitive field effect transistor (ISFET). Application of the methodology on enzyme preparations showed that the sensor is able to detect carbapenemases of the NDM, IMP, KPC and NMC-A types at low nanomolar concentrations while VIM and OXA-48 responded at levels above 100 nM. Similar results were obtained when CPE cell suspensions were tested; NDM, IMP, NMC-A and KPC producers caused fast reductions of the output potential. Reduction rates with VIM-type and especially OXA-48 producing strains were significantly lower. Based on results with selected CPEs and carbapenemase-negative enterobacteria, a threshold of 10 mV drop at 30 min was set. Applying this threshold, the method exhibited 100% sensitivity for NDM, IMP and KPC and 77.3% for VIM producers. The OXA-48-positive strains failed to pass the detection threshold. A wide variety of carbapenemase-negative control strains were all classified as negative (100% specificity). In conclusion, an ISFET-based approach may have the potential to be routinely used for non OXA-48-like CPE detection in the clinical laboratory.

## Introduction

The ongoing spread of multidrug and carbapenemase-producing *Enterobacterales* (CPE), primarily *Klebsiella pneumoniae*, has been recognized by WHO and other international institutions as a major public health problem. CPE infect hospitalized patients worldwide and, given the limited therapeutic options, may result in unacceptably high mortality rates especially among debilitated patients treated in ICUs. Carbapenemases encountered in clinical enterobacteria comprise a variety of evolutionary distinct β-lactamase types of unknown origin the most common being the serine reactive KPC variants (class A), the zinc-dependent metallo-β-lactamases (MβLs) of the NDM, VIM, and IMP types (class B), and the serine reactive OXA-48 enzyme (class D). The respective genes are invariably associated with promiscuous genetic structures that facilitate further dissemination^1–6^.

Successful containment of CPEs through implementation of appropriate infection control measures seems, at least currently, as the main way to limit the serious burden posed by these pathogens on health systems. The task of containment falls mainly with the microbiology laboratory that should be able to detect these strains in an accurately and timely fashion. Carbapenemase production is not always associated with increased carbapenem MICs^7^. Therefore several such strains may evade a laboratory that solely relies in antimicrobial susceptibility testing (AST) for detection of carbapenemase producers. Molecular methods and immunochromatography can provide the required high sensitivity but again would fail to detect the gene or production of a novel carbapenemase^8–10^. Phenotypic techniques based on synergy tests using carbapenem AST discs that contain inhibitory molecules can provide reliable results not only on the production of even novel enzymes but regarding their type as well (e.g. serine reactive or MβL;^11^). Carbapenemase production may as well be revealed indirectly by carbapenem inactivation assays using a sensitive indicator strain (e.g. modified Hodge test;^12^). A definite answer on whether a bacterium produces a carbapenemase or not may be provided by monitoring hydrolysis of a carbapenem through UV spectrophotometry using crude protein extracts^13^. Enzymatic assays though, are not preferred in a clinical laboratory, due to the additional step of protein extraction and the required specialized equipment. Mass spectrometric profiling of the carbapenem hydrolysis products has also being employed utilizing the MALDI-TOF instruments used for bacterial species identification^14,15^.

**Figure 1:**
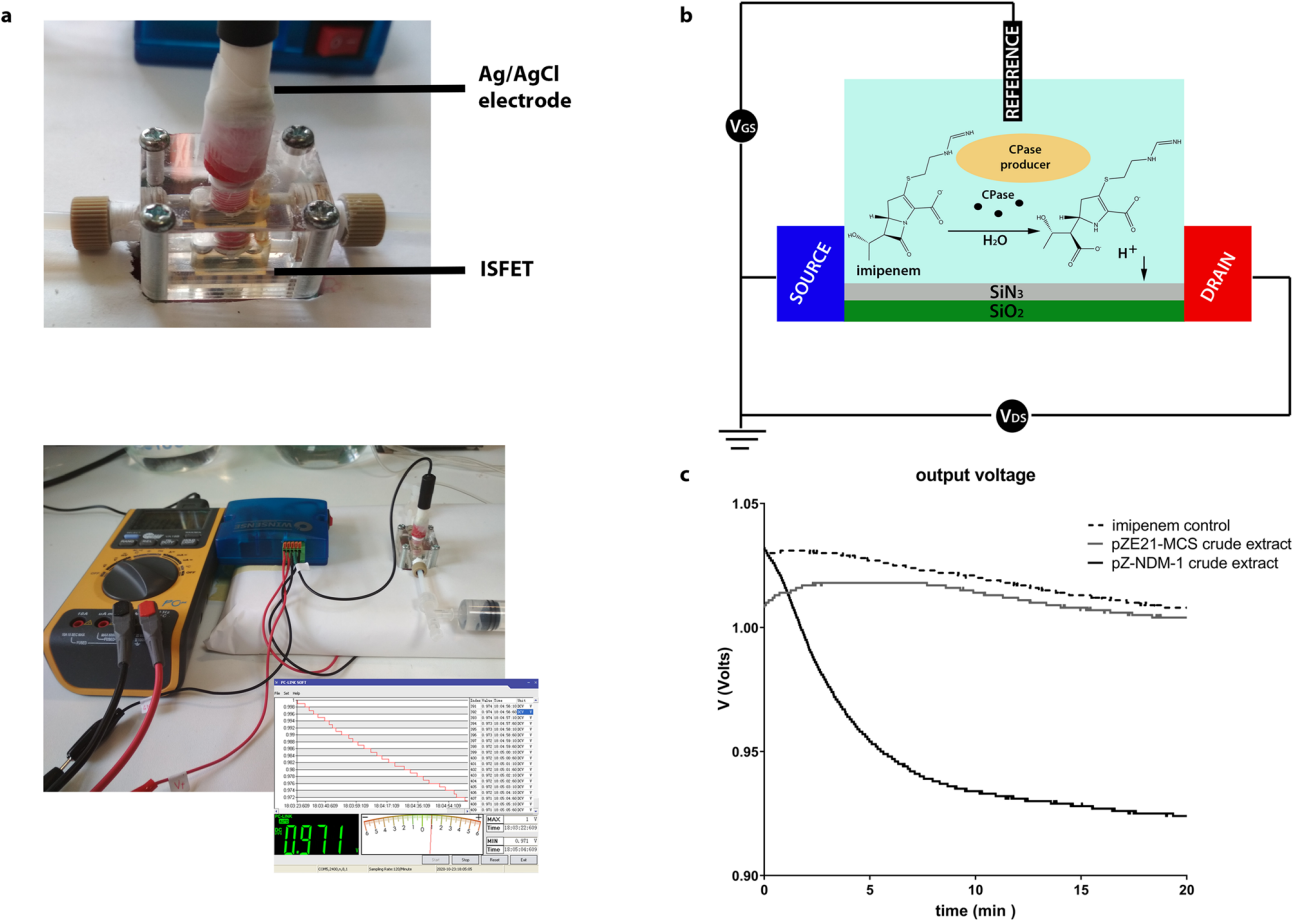
(**a**) Equipment used in the study. The ISFET was placed in the bottom of a flow cell atop of which the Ag/AgCl reference electrode is located in contact with the solutions applied to the apparatus. A stable current and voltage was provided to the sensor by a commercially available circuit and the difference in potential was measured with a PC connected multimeter. (**b**) Diagram of the ISFET and principle of the method. The gate insulator material can accept and donate protons. Any change in the hydrogen cation concentration of the solution, e.g. due to hydrolysis of imipenem by a carbapenemase (CPase), will modify the equilibrium yielding changes of the potential across the transistor causing proportional shifts of the output voltage. (**c**) Changes in output voltage caused by 6.67 mg/ml imipenem alone (imipenem control) or containing equal quantities of crude protein extracts of *E. coli* clones (56 μg total protein content) carrying the pZE21-NDM-1 recombinant plasmid or the pZE21-MCS vector, monitored for 20 min. The voltage reduction caused by the NDM-1 mediated imipenem hydrolysis is apparent.

Detection of the acidic hydrolysis product (Fig. 1b) of carbapenems by pH indicators is a promising alternative as there is no need for specialized equipment and laborious protein extraction^16^. The acidimetric determination of β-lactam hydrolysis has been a method introduced more than 60 years ago in enzymology studies of penicillinases^17^. Nordman et al. developed a modification of the technique that uses imipenem as a substrate and therefore could detect production of an enzyme with carbapenemase activity^18^. The technique was subsequently commercialized (CARBA NP) while a similar derivative that uses a different pH indicator and modifications that do not require cell disruptive reagents has been developed (Blue-Carba;^19^). Electrochemical detection of the protons produced during imipenem hydrolysis by CPE has been also employed using a polyaniline coated pH-redox sensor with its performance being comparable to that of the pH indicator techniques (BYE Carba;^20,21^).

Theoretically, potentiometric detection of the protons produced during imipenem hydrolysis by carbapenemases could also be carried out using an Ion Sensitive Field Effect Transistor (ISFET) sensor. ISFETs were the first chemical sensors to be developed 50 years ago^22^ and are actually Metal Oxide Semiconductor Field Effect Transistors (MOSFETs) where the metal gate has been substituted with a reference electrode submerged in the aqueous solution interfacing the metal oxide^23^. When the gate insulator material can buffer specific ions and the drain current is stable, changes in the potential across the capacitor (*ΔΨ*) would be analogous to the changes of the ions’ concentration^23^. In pH sensing ISFETs the gate insulator is made of amphoteric materials such as silicon nitride (Si_3_N_4_). There, in the solution/transistor interface, the following equilibria are being established^24^:

1 $${\text{SiOH }}\mathop{\rightharpoonup}\limits_{\leftharpoondown} {\text{SiO}}^{ - } + {\text{H}}^{ + }$$

2 $${\text{SiOH}}_{{2}}^{ + } \mathop{\rightharpoonup}\limits_{\leftharpoondown}{\text{SiOH }} + {\text{ H}}^{ + }$$

3 $${\text{SiNH}}_{{3}}^{ + } \mathop{\rightharpoonup}\limits_{\leftharpoondown} {\text{SiNH}}_{{2}} + {\text{ H}}^{ + }$$

With the pH changes in the solution controlling the changes in the potential according to the following equation^23^:

4 $$\Delta \Psi = - 2.3\alpha \frac{RT}{F}\Delta pH$$

where *R* is the universal gas constant, *F* the Faraday constant, *T* the temperature and *α* the sensitivity factor of the gate insulator material (usually < 1) that when approaching unity the response of the transistor equals the sensitivity predicted by the Nernst equation (58.2 mV per pH unit change at 20 °C).

Due to their ease in construction, low cost, reusability and low volume, ISFET sensors have been exploited in a variety of applications not only as sensitive and fast response pH meters but also in more advanced methodologies requiring detection of the protons produced during biochemical reactions^23,25^. Replacement of the conventional Ag/AgCl reference electrode by coupling the ISFET with another field effect transistor (REFET;^26,27^) permitted further volume reductions and the development of microchips such as those used in the IonTorrent semiconductor DNA sequencing technology^28^. Manufacturing of circuits that would amplify the signal to well above the Nernstian response enabled the detection of pH shifts during growth of *E. coli* at densities as low as 10^2^ cfu/ml^24^. Herein we explore the applicability of such a sensor to detect imipenem hydrolysis by free carbapenemases and suspensions of CPE cells using a low cost commercially available flow cell ISFET with a conventional reference electrode.

## Results

### Application of the ISFET method on β-lactamase preparations

**Figure 2:**
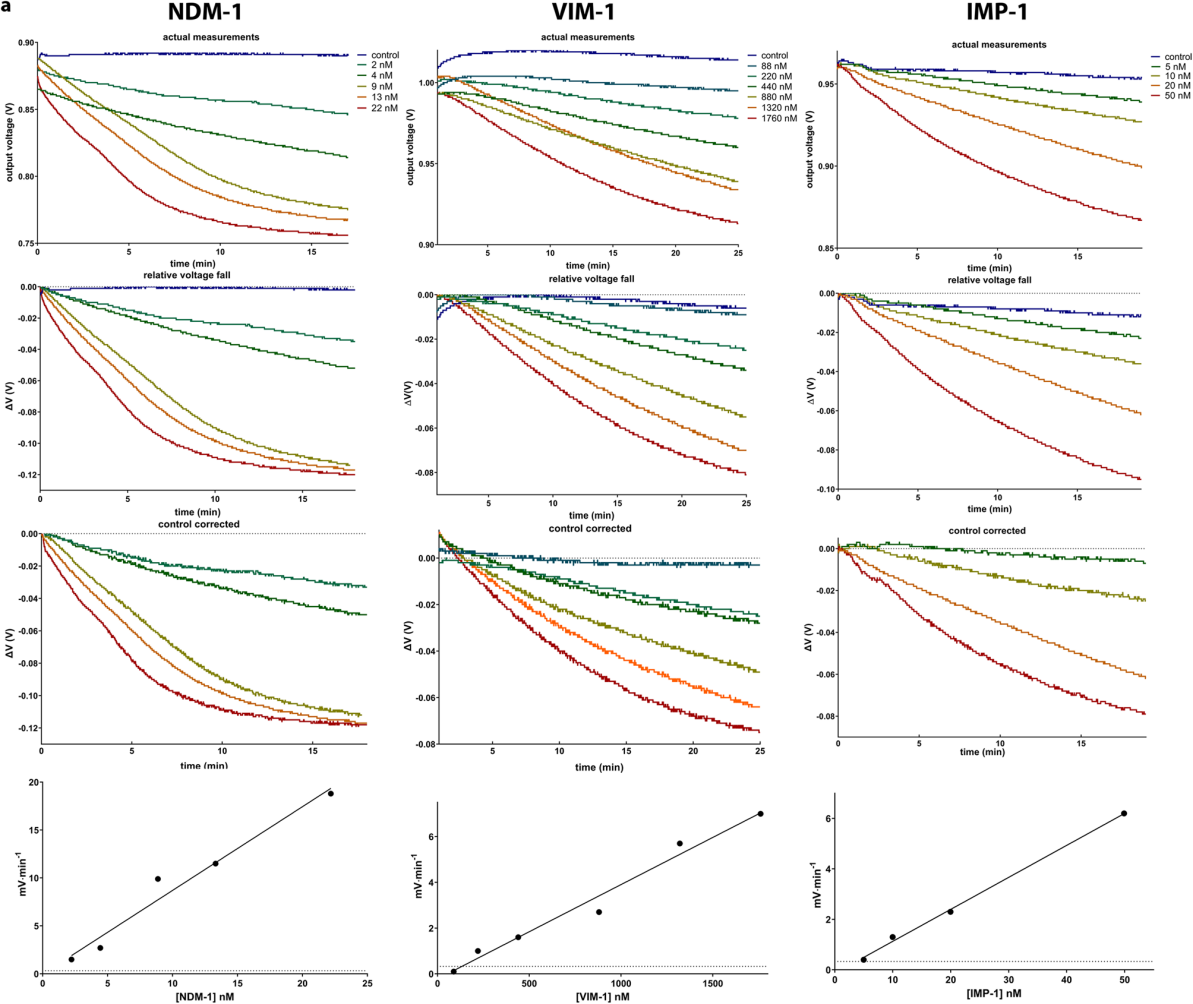

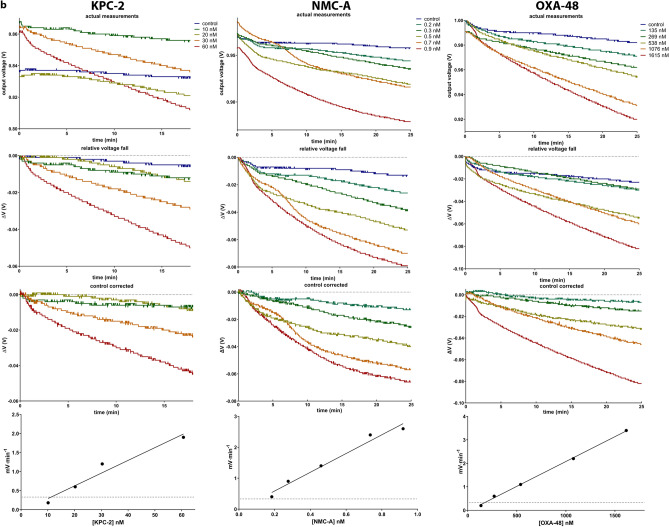
ISFET measurements using various quantities of MβL (**a**) and serine reactive (SbL) carbapenemase (**b**) containing crude protein extracts. The enzyme concentration in each preparation was estimated through spectrophotometric measurement of imipenem hydrolysis and the *Michaelis–Menten* equation using the published steady-state constants. Graphs of the actual measurements, of relative voltage changes and of control corrected curves are shown. For the linear phase of each corrected curve the rate of voltage change was determined and plotted against the enzyme quantity (lower graphs). The detection threshold was set to − 0.33 mV/min and the enzyme concentration required to reach this limit was estimated through linear regression.

The ability of the ISFET method in detecting imipenem hydrolysis was assessed using β-lactamase preparations obtained from recombinant *E. coli* clones. Initial experiments were performed using protein extracts from the *E. coli* pZ-NDM-1 clone and from *E. coli* carrying the empty vector pZE21-MCS. Addition of the NDM-1 containing preparation in the imipenem solution caused a significant drop in the output voltage of the apparatus, in contrast to equal quantities of the preparation obtained from the pZE21-MCS which yielded rates that were similar with the imipenem control (Fig. 1c). In the NDM-1/imipenem reaction mix the voltage changed linearly with time until a fall of approximately 90–100 mV where the rate started to slow reaching a plateau at 120 mV (corresponded to approx. 2.7 pH units’ fall i.e. the pH fell from 7.2 to 4.5) probably due to the lower enzyme activity and stability at the acidic pH. The rate of voltage reduction, estimated from control corrected curves, was analogous to the quantity of NDM-1 added in the reactions (Fig. 2a). Similar patterns were observed for the VIM-1 and IMP-1 MβLs (Fig. 2a) as well as for the KPC-2, NMC-A and OXA-48 serine reactive carbapenemases (Fig. 2b). The CTX-M-15 and CMY-2 β-lactamases, not hydrolyzing imipenem, exhibited a relative voltage reduction near zero as compared to control.

The responses of the sensor to the various types of carbapenemases differed with some enzymes requiring higher quantities in order to observe measurable pH changes (Fig. 2a,b). The efficiency of the ISFET sensor to detect imipenem hydrolysis by the various carbapenemase types was estimated through comparisons of the rate of voltage reduction per one microliter of crude extract (*V*_ISFET_) with the rate of absorbance units fall at 300 nm (*V*_UV_) caused by the same preparation during spectrophotometric monitoring of 80 μM imipenem hydrolysis in the ZnSO_4_ solution in parallel experiments (Table 1). The *V*_ISFET_/*V*_UV_ ratios indicated that from the tested β-lactamases NDM-1 could be detected with higher efficiency followed, in descending order, by NMC-A, IMP-1, KPC-2, VIM-1 and OXA-48 (Table 1). Moreover, comparisons of the lowest concentrations of the enzymes that could be detected with the ISFET (using a threshold of 10 mV fall at 30 min) showed that detection limits were at the sub-nanomolar level for NMC-A and NDM-1, nanomolar for IMP-1 and KPC-2, and sub-micromolar level for VIM-1 and OXA-48 (Table 1; Fig. 2). The ISFET detection efficiency for the various carbapenemases correlated with the respective steady-state catalysis constants (*k*_cat_) with enzymes exhibiting rapid imipenem turn-over causing higher pH shifts (Table 1).

**Table 1:** Imipenem hydrolysis monitored through the ISFET sensor and UV spectrophotometry using crude enzyme preparations.

Preparation	*V*_*I*SFET_	*V*_UV_^a^	*V*_ISFET_/*V*_UV_	ISFET detection limit	*k*_cat_^c^	*K*_m_^c^
	(mV·min^−1^·μl^−1^)	(AU·min^−1^·μl^−1^)	(mV·AU^−1^)	(nM)^b^	(sec^−1^)	(μM)
VIM-1	− 0.18 ± 0.02	− 0.070 ± 0.001	2.5	130	2	1.5
NDM-1	− 1.9 ± 0.2	− 0.080 ± 0.001	24	0.4	64	35
IMP-1	− 1.3 ± 0.1	− 0.25 ± 0.04	5.2	4	46	39
KPC-2	− 0.007 ± 0.001	− 0.0015 ± 0.0003	4.7	11	15	51
NMC-A	− 2.8 ± 0.3	− 0.36 ± 0.05	7.8	0.1	1040	92
OXA-48	− 0.011 ± 0.002	− 0.018 ± 0.003	0.6	172	4.8	13

^a^Data represent means ± standard deviations of three measurements.

^b^Approximate enzyme concentration in the final measurement solution.

^c^Data published in references 39–44.

*V*_ISFET_: Voltage fall per minute per μl of enzyme preparation used.

UV: absorbance units fall at 300 nm during hydrolysis of 80 μM imipenem in the ZnSO_4_ solution per minute per μl of enzyme preparation used.

### Whole cell assays using laboratory *E. coli* clones

**Figure 3:**
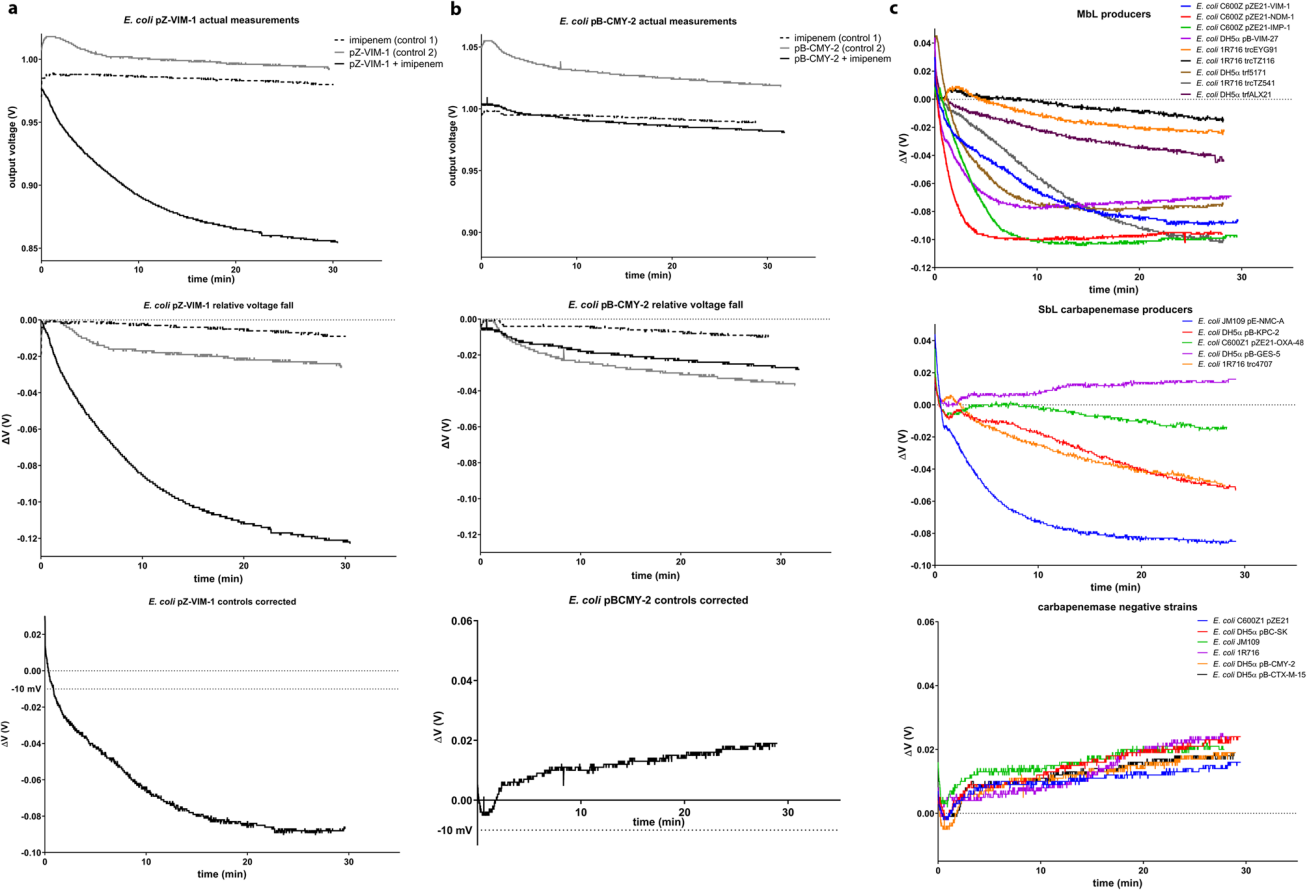
Assays using laboratory *E. coli* strains directly from bacterial cultures. (**a,b**)**:** Measurements of clones overproducing the VIM-1 MβL and the CMY-2 class C beta-lactamase. In each experiment three measurements were obtained corresponding to the imipenem solution (control 1), the bacterial suspension (control 2) and their between mixture. The normalized curve of the imipenem-bacterium mix was corrected by subtracting those of the two controls. (**c**) Cumulative data of laboratory strains experiments. Control corrected curves of bacterial suspensions when interacting with imipenem are shown. MβL as well as the majority of SbL carbapenemase producing clones yielded imipenem hydrolysis that was detectable by the ISFET. The strain overproducing the GES-5 carbapenemase and the clones expressing β-lactamases with weak or no imipenemase activity yielded voltage changes in the corrected curves that were greater than zero.

Preliminary experiments showed that the apparatus was able to detect imipenem hydrolysis caused by dense suspensions of intact MβL producing recombinant *E. coli* clones (e.g. *E. coli* pZE21-VIM-1; Fig. 3a). The voltage fall observed with the mixture of the bacterial suspensions and imipenem was considerably higher compared to either the bacterial suspension alone or imipenem (Fig. 3a). Yet, in the measurements of bacterial suspensions (Fig. 3; control 2 curves) the pH was not stable and after an initial increase phase it dropped at rates higher than the imipenem measurement (Fig. 3; control 1 curves). This was rather anticipated, as experiments were performed in un-buffered conditions and intact bacterial cells could affect the ion composition of their environment. The above observations suggested that in experiments of whole bacterial cells an additional control measurement of the bacterial suspension alone had to be included and that the bacterium/imipenem curve had to be corrected by subtracting both control curves (Fig. 3a; lower graph). Application of the ISFET method on recombinant clones over-producing non-carbapenemases (e.g. *E. coli* pBCMY-2; Fig. 3b) revealed that the voltage fall observed with the bacterium/imipenem mixtures was lower compared with that of the bacterium alone with the latter measurements yielding even higher rates of pH changes (probably due to a buffering effect of the imipenem solution), thus suggesting that any minor imipenem hydrolysis by non-carbapenemase producing bacteria could not be detected by the apparatus.

The above results provided strong evidence that the apparatus could differentiate bacteria producing carbapenemases from those that did not. To assert this, additional recombinant *E. coli* clones overproducing various β-lactamases (i.e. NDM-1, IMP-1, VIM-27, KPC-2, NMC-A, OXA-48, GES-5 and CTX-M-15) as well as, laboratory generated *E. coli* clones harboring wild type carbapenemase encoding plasmids, were analyzed. *E. coli* hosts as well as clones carrying empty plasmid vectors were also included in the experiments. The corrected voltage—time curves of bacteria/imipenem reactions showed that the total of MβL producers induced significant reductions of the apparatus readings, while from the strains producing serine reactive carbapenemases only NMC-A, KPC-2 and OXA-48 exhibited a voltage drop (Fig. 3c). The GES-5 producing strain yielded a positive corrected voltage change indicating that the low hydrolysis rates conferred by this enzyme could not be detected by the method (Fig. 3c; medium graph). Similarly to the GES-5 clone, the strains not producing a carbapenemase yielded a voltage fall that was lower compared to that of the controls (Fig. 3c; lower graph).

Among the carbapenemase overproducing clones the pZ-OXA-48 induced the lowest responses averaging a drop of 16 mV at 30 min while the rest exhibited a voltage reduction above 50 mV (Table 2). Expression of VIM-1, KPC-2 and NDM-1 by natural plasmids in *E. coli* was detectable by the ISFET sensor in all cases (Table 2). Here, the VIM-1 producing clones gave the weakest responses (as low as 22 mV; Table 2). The majority of efficient carbapenemase producers passed the threshold of − 10 mV in less than 5 min with the exceptions of pZ-OXA-48 clone and the VIM-1 expressing *E. coli* 1R716/trc-TZ116 transconjugant (14 and 18 min respectively; Table 2). The magnitude of voltage changes in carbapenemase producing *E. coli* laboratory strains did not correlate with the respective carbapenem resistance levels as indicated by imipenem and meropenem MICs (Table 2).

**Table 2:** ISFET detection of imipenem hydrolysis by laboratory generated *E. coli* clones.

Host/plasmid	β-lactamases	ΔV_30 min_	t_ΔV < − 10 mV_	MIC (μg/ml)	
		(mV)^a^	(min)	Imipenem	Meropenem
***E. coli* hosts/vectors**					
DH5a/pBC-SK	none^b^	> 0	NA	0.25	< 0.125
C600Z1/pZE21	none	> 0	NA	0.25	< 0.125
1R716	none	> 0	NA	0.25	< 0.125
JM109	none	> 0	NA	0.25	< 0.125
***E. coli*** **hosts/Recombinant plasmids**					
DH5a/pB-bla_VIM-27_	VIM-27	− 68 ± 15	< 1	4	1
C600Z1/pZ- bla_VIM-1_	VIM-1	− 90 ± 10	2 ± 1	2	1
C600Z1/pZ- bla_NDM-1_	NDM-1	− 92 ± 5	< 1	64	64
C600Z1/pZ- bla_IMP-1_	IMP-1	− 96 ± 2	2 ± 1	16	32
DH5a/pB-bla_KPC-2_	KPC-2	− 52 ± 9	3 ± 1	8	1
JM109/pΝ- bla_NMC-A_	NMC-A	− 84 ± 6	< 1	32	4
C600Z1/pZ- bla_OXA-48_	OXA-48	− 16 ± 4	14 ± 6	1	0.5
DH5a/pB-bla_GES-5_	GES-5	> 0	NA	1	0.25
DH5a/pB-bla_CMY-2_	CMY-2	> 0	NA	0.25	< 0.125
DH5a/pB-bla_CTX-M-15_	CTX-M-15	> 0	NA	0.25	< 0.125
***E. coli* hosts/Natural plasmids**					
1R716/trc-TZ116	VIM-1/CMY-13	− 22 ± 3	18 ± 2	4	1
1R716/trc-TZ541	VIM-1/CMY-13	− 95 ± 8	1	8	2
RC85/trc-EUG91	VIM-1/VEB-1/OXA-10	− 26 ± 5	5 ± 4	16	0.5
1R716/trc-ALX21	VIM-1/VEB-1	− 46 ± 10	4 ± 2	ND	ND
1R716/trc4707-KPC-2	KPC-2	− 53 ± 6	4 ± 1	16	8
DH5a/trf5171	NDM-1	− 77 ± 15	2 ± 1	2	1

^a^Means of three measurements are shown ± standard deviations.

^b^*E. coli* hosts carried the chromosomal class C β-lactamase expressed at basal levels.

ND: Not determined, NA: Not applicable.

### Application on clinical isolates

The ISFET method was subsequently challenged against a collection of carbapenemase positive or negative clinical enterobacterial isolates (Tables 3, 4). Production of an MβL or KPC-2 was detectable by the sensor in all of the *K. pneumoniae* strains tested (Table 3). In contrast none of the OXA-48 producing strains and the GES-6 isolate induced a voltage reduction within the time course of monitoring. Positive corrected voltage changes were also obtained in the experiments of the carbapenemase negative strains. The strains producing NDM, IMP and KPC type carbapenemases gave the strongest signals while for the VIM-producing isolates a range of responses was observed with some strains yielding a voltage fall near the threshold of 10 mV.

**Table 3:** Application of the ISFET method on clinical isolates.

Isolate	β-lactamases	ΔV_30 min_ (mV)	t_ΔV < − 10 mV_ (min)	MIC (μg/ml)	
				Imipenem	Meropenem
**CPase positive *K. pneumoniae***					
SEC-4	VIM-1	− 39	7	64	64
IK-14	VIM-1/CMY	− 11	29	1	0.5
ESDY-2681	VIM-27	− 24	2	8	32
UoA-17	VIM-1	− 36	2	32	32
HIP-646	VIM-1/SHV-5	− 17	10	32	32
TZ-52/531	VIM-1	− 18	8	4	2
UA-12/227	VIM-1	− 29	5	8	2
UA-1955	VIM-1	− 41	8	4	2
LA-30	VIM/CMY	− 71	< 1	16	16
EYG-912	VIM-1	− 27	1	16	8
LA-26	NDM-1/CTX-M	− 43	< 1	16	32
LA-27	NDM-1/CTX-M	− 72	< 1	8	8
LA-28	NDM-1/CTX-M	− 58	< 1	8	64
2489	NDM-1/CTX-M	− 79	2	32	64
LA-419/15	NDM-1/CTX-M	− 46	3	8	32
LAR-26I/15	NDM/VIM/CTX-M	− 66	< 1	16	64
LAR-37II/15	NDM/VIM/CTX-M	− 63	2	32	32
LAR-38I	NDM/VIM/CTX-M	− 58	< 1	8	16
ESDY-5742	IMP-1	− 83	1	16	16
E-1370	KPC-2	− 64	2	2	4
E-1253	KPC-2/SHV-12	− 46	3	32	32
E-1505	KPC-2	− 56	2	32	16
E-1516	KPC-2/SHV-12	− 79	2	16	8
E-1782	KPC-2	− 71	2	2	16
E-1810	KPC-2	− 65	2	32	32
ΕUG-971	KPC-2/SHV-5	− 80	3	1	8
E-1797	KPC-2/VIM-19/CMY	− 70	1	1	2
EYG-47	KPC-2/VIM-1/SHV	− 89	< 1	32	32
EYG-240	KPC-2/VIM-1/SHV	− 90	< 1	8	16
ALX-47	OXA-48/CTX-M	> 0	NA	4	8
LA-478	OXA-48/CTX-M	> 0	NA	8	8
TRK-2	OXA-48/CTX-M	> 0	NA	32	64
TRK-1	OXA-48/CTX-M	> 0	NA	8	32
TRK-5	OXA-48/CTX-M	> 0	NA	16	32
LA-3878	GES-6/SHV-5	> 0	NA	16	32
**CPase negative *K. pneumoniae***					
17,829	CTX-M-15/SHV-12	> 0	NA	8	16
17,830	CTX-M-15/SHV-12	> 0	NA	0.25	2
71,697	CTX-M-15/OXA-1	> 0	NA	0.25	1
729B	CTX-M-15/SHV-11	> 0	NA	0.25	2
EY-205	CMY-36/SHV-5	> 0	NA	0.5	< 0.125
3996	CMY-36/SHV-5	> 0	NA	0.25	< 0.125
T80	LAT-2	> 0	NA	0.25	< 0.125
4698	SHV-5	> 0	NA	0.25	1
IpT-58	GES-7	> 0	NA	0.5	< 0.125
IpT-59	GES-7/SHV-5	> 0	NA	0.25	< 0.125
TZ-59	species specific SHV	> 0	NA	0.25	< 0.125
6478/Α	species specific SHV	> 0	NA	0.25	< 0.125
**CPase positive** ***Enterobacterales*** (**other than *K. pneumoniae***)					
***E. coli***					
TZ116	VIM-1/CMY-13	− 14	27	4	2
TZ541	VIM-1/CMY-13	− 81	2	8	2
LAR82	KPC-2/OXA-1/CTX-M	− 67	4	4	1
LAR148	KPC-2/OXA-1/CTX-M	− 61	2	4	1
LAR152	KPC-2/OXA-1/CTX-M	− 71	1	2	1
LAR373	KPC-2	− 106	1	4	1
LAR548	KPC-2	− 101	2	32	64
TZ3638	GES-5	> 0	NA	0.5	0.25
***Enterobacter cloacae***					
SEC-5	VIM-1	− 13	28	32	8
SEC-6	VIM-1	− 8	NA	1	0.5
***Proteus mirabilis***					
EYG91	VIM-1/VEB-1	− 10	30	4	< 0.125
EYG92	VIM-1/VEB-1	− 5	NA	4	< 0.125
ESDY-11173	VIM-1/CMY-16	− 2	NA	0.5	1
ESDY-15184	VIM-1/CMY-16	> 0	NA	2	< 0.125
ESDY-2530	VIM-1/CMY-16	> 0	NA	4	< 0.125
***Providencia stuartii***					
EYG323	VIM-1	− 81	1	8	0.5
ALX21	VIM-1/VEB-1/OXA-10	− 30	10	8	2
***Serratia liquefaciens***					
EYG815	VIM-1/SHV-5	− 66	5	32	32
**CPase negative ** ***Enterobacterales***(**other than *K. pneumoniae***)					
*E. coli* IK33	CTX-M-15	− 5 ± 2	NA	0.25	≤ 0.125
*E. coli* S13	CTX-M-32	> 0	NA	0.25	≤ 0.125
*E. coli* S80	CTX-M-15/GES-7	> 0	NA	0.25	≤ 0.125
*E. coli* TZ27	CTX-M-3/LAT-2	> 0	NA	1	1
*E. coli* MEL1	LAT-3	> 0	NA	0.5	≤ 0.125
*E. coli* MEL2	LAT-4/SHV-5	> 0	NA	1	≤ 0.125
*E. coli* EY 03	Chr. AmpC	> 0	NA	0.25	≤ 0.125
*E. coli* EY 157	Chr. AmpC	> 0	NA	0.25	≤ 0.125
*E. coli* EY 164	Chr. AmpC	> 0	NA	0.25	≤ 0.125
*E. coli* EY 165	Chr. AmpC	> 0	NA	0.25	≤ 0.125
*K. aerogenes* EY-15	LAT-2	> 0	NA	8	2
*K. aerogenes* EY-25	LAT-2/SHV-5	> 0	NA	16	8
*E. cloacae* TSV9	GES-7	> 0	NA	0.5	≤ 0.125
*K. aerogenes* EY-171	Derepr.AmpC	> 0	NA	4	0.25
*E. cloacae* EY 138	Derepr.AmpC	> 0	NA	0.5	≤ 0.125
*K. aerogenes* EY 13	Derepr.AmpC	− 2 ± 3	NA	0.25	≤ 0.125
*E. cloacae* EY 168	Ind. AmpC/TEM-1	> 0	NA	0.5	≤ 0.125
*P. mirabilis* AT20	CTX-M-15	> 0	NA	0.5	0.5

**Derepr. AmpC:** De-repressed expression of the chromosomal cephalosporinase, **Ind. AmpC:** Inducible expression of the chromosomal cephalosporinase, **Chr. AmpC:** Basal expression of the chromosomal cephalosporianase, **NA:** Not applicable.

**Table 4:** Assessment of ISFET false negative results through prolonged measurements and comparisons with RAPIDEC CARBA NP (ISFET true positives and true negatives included).

Isolate	β-lactamases	ISFET result (at 30 min)	ΔV_80 min_ (mV)	t_ΔV < − 10 mV_ (min)	CARBA NP result/color
*E. coli* LAR548	KPC-2	Positive	NP	1	Positive/Yellow
*K. pneumoniae* LA28	NDM-1/CTX-M	Positive	NP	< 1	Positive/Yellow
*K. pneumoniae* LA30	VIM/CMY	Positive	NP	< 1	Positive/Yellow
*P. mirabilis* 2530	VIM-1/CMY-16	Negative	− 17	62	Positive/Orange
*P. mirabilis* 11,173	VIM-1/CMY-16	Negative	NP	NA	Positive/Yellow
*P. mirabilis* EYG92	VIM-1/CMY-16	Negative	NP	NA	Positive/Orange
*K. pneumoniae* LA-47	OXA-48/CTX-M	Negative	− 60	45	Positive/Orange
*K. pneumoniae* LA-478	OXA-48/CTX-M	Negative	− 28	51	Positive/Orange
*K. pneumoniae* TRK-1	OXA-48/CTX-M	Negative	− 2	NA	Negative/Red
*K. pneumoniae* TRK-2	OXA-48/CTX-M	Negative	− 12	76	Positive/Orange^a^
*K. pneumoniae* TRK-5	OXA-48/CTX-M	Negative	− 5	NA	Negative/Red
*K. pneumoniae* EY-205	CMY-36/SHV-5	Negative	− 2	NA	Positive/Orange^a^
*K. pneumoniae* 17,829	CTX-M-15/SHV-12	Negative	5	NA	Positive/Orange^a^
*K. aerogenes* EY-25	LAT-2/SHV-5	Negative	15	NA	Negative/Red
*E. cloacae* EY 138	Derepr. AmpC	Negative	NP	NA	Negative/Orange^b^

NP: Not performed.

NA: Not applicable as the − 10 mV threshold had not been reached.

^a^Result became positive after 2 h of incubation at 37 °C.

^b^The control well lacking imipenem yielded also an orange color.

Similar results regarding VIM detection were obtained when analyzing non *K. pneumoniae* enterobacterial strains (Table 4). In *E. coli* VIM production was detectable in both isolates tested with one strain inducing a weak (*E. coli* TZ116) and the other a strong response (*E. coli* TZ541). As the same difference was observed for the *E. coli* transconjugants of the above strains (Table 2) it was more likely due to the lower expression of the MβL gene by the TZ116 VIM carrying plasmid. A weak acidification was also evident in the two *E. cloacae* VIM-1 producers analyzed with one of them not exceeding the threshold of 10 mV at 30 min (Table 4). Detection of this MβL was moreover problematic in the five *P. mirabilis* isolates tested with only one of them reaching the threshold and two of them exhibiting a positive corrected voltage change. In contrast, VIM-1 production in *P. stuartii* and *S. liquefaciens* induced significant pH reduction. As was observed with *K. pneumoniae* isolates, KPC-2 producing *E. coli* caused rapid voltage changes in all isolates while the strain expressing the less efficient class A carbapenemase GES-5 did not yield a measurable, by the ISFET sensor, imipenem hydrolysis. In carbapenemase negative *E. coli*, *E. cloacae*, *E. aerogenes* and *P. mirabilis* isolates a greater than zero corrected voltage change was observed in all but two cases where a minor decrease, below 5 mV, was documented (Table 4).

Based on the above data, a voltage reduction of 10 mV in 30 min in the control corrected curve would indicate production of an efficient carbapenemase (in terms of k_cat_/K_m_) by the analyzed strain. Application of this threshold to the dataset of clinical isolates examined herein would identify 42 out of the 53 enterobacterial carbapenemase producers (79.2% sensitivity) while it would exclude all the 30 strains not producing such an enzyme as negative (100% specificity). Regarding specific carbapenemase types the ISFET could detect 100% of the NDM-1, KPC-2 and IMP-1 producers and 17 out of 22 strains expressing solely a carbapenemase of the VIM type (77.3% sensitivity) while failing to identify any of the OXA-48 and GES type carbapenemase strains. The relatively low sensitivity in detecting VIM producers was confined to *E. cloacae* (with 1 out of 2 strains yielding a false negative result) and *P. mirabilis* (4/5 false negatives).

In a number of OXA-48 K*. pneumoniae* measurements it was apparent that the control corrected relative voltage, though positive, had a downward trend indicating that the 30 min time window may not be adequate to reveal imipenem hydrolysis by this enzyme when expressed by natural promoters. In order to assess this, additional measurements were performed where the voltage was monitored for 80 min. In three out of the five OXA-48 strains the − 10 mV detection limit was reached between 45 and 76 min (Table 4). Voltage monitoring for extended periods may also enable the detection of VIM producing *P. mirabilis*, as indicated by prolonged measurements for one such isolate (Table 4). The fact that from the five VIM producing *P. mirabilis* the apparatus could detect imipenem hydrolysis only in one isolate (i.e. opposite to detection rates observed for this enzyme in the other enterobacterial species) suggested that there may be expression issues of the enzyme in these species. This was indicated by the fact that the VIM producing *E. coli* trc-EUG91 clone obtained through conjugation with *P .mirabilis* EUG91 exhibited stronger ISFET responses compared to the donor. In order to assess if this was indeed caused by lower VIM production and not due to other species specific factors (e.g. cell wall endurance), protein extracts of *P. mirabilis* EUG91 and its *E. coli* transconjugant were assayed for imipenem hydrolysis using spectrophotometry. The measured imipenem hydrolysis units (33 nmol∙min^−1^∙mg^−1^ for *P. mirabilis* versus 136 nmol∙min^−1^∙mg^−1^ for *E. coli*) indicated that the enzyme was produced at lower levels in the periplasm of *P. mirabilis* thus causing difficult to resolve detection issues.

Additional indications that the ISFET false negative results for OXA-48 and VIM producers were due to low enzyme levels in the measurement solution were obtained through comparisons with the commercial colorimetric method RAPIDEC CARBA NP. The latter test uses denser bacterial suspensions than those used in ISFET (due to lower reaction volume) with the measurement being preceded by a 30 min bacterial cell lysis step. CARBA NP enabled the detection of three out of five OXA-48 producers while it failed to identify two isolates with results being in accordance with prolonged ISFET measurements (Table 4). Regarding VIM producing *P. mirabilis* CARBA NP identified all the three isolates tested (Table 4). With the exception of one *P .mirabilis* strain that yielded a yellow color, all the ISFET false negative strains tested with CARBA NP did not cause significant pH shifts as revealed by the orange color of the indicator (Table 4). Though the colorimetric method was more sensitive as compared to the ISFET it suffered from specificity issues as two out of the four carbapenemase negative *Enterobacterales* strains tested, which were overproducing CTX-M-15 and CMY-2 type beta-lactamases, yielded a positive result (Table 4). In contrast, the ISFET method, even in prolonged measurements, did not yield any false positive result (Table 4).

## Discussion

**Figure 4:**
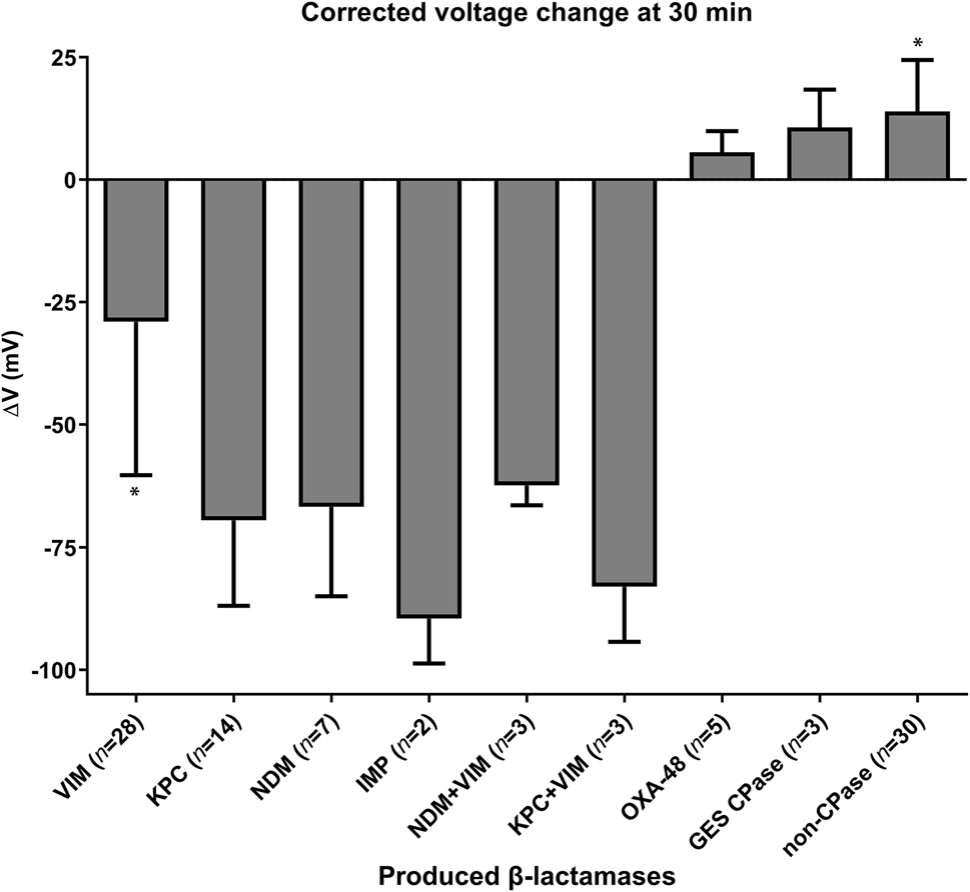
Voltage changes observed during 30 min measurements of the various laboratory and clinical strains assayed in this study grouped according to the β-lactamase type produced. NDM, IMP and KPC carbapenemase producing enterobacteria yielded the highest pH reductions while VIM strains exhibited mixed performance. The 30 min time window was not adequate to reveal imipenem hydrolysis by OXA-48 K*. pneumoniae* as the voltage changes were positive similarly with the producers of the weak imipinemases of the GES type (i.e. GES-5 and GES-6) and carbapenemase negative strains. Comparison between the VIM producing and the non-CPase data sets using unpaired t-test yielded a two-tailed *p* value < 0.0001 (*).

Herein we assessed the ability of a commercially available ion sensitive field effect transistor sensor to detect the protons produced when imipenem is hydrolyzed by clinically relevant carbapenemases. Application of the method on β-lactamase preparations and whole cells of various enterobacterial species indicated that the ISFET could detect most of the clinically relevant carbapenemases with high specificity and relatively high sensitivity. Enzymes exhibiting low turn-over rates during imipenem hydrolysis (e.g. OXA-48 and VIM-1) required higher quantities in order to be detected under the experimental conditions tested (i.e. at an imipenem concentration well above the respective *K*_m_s) compared to the faster carbapenemases (NDM-1, NMC-A, IMP-1 and KPC-2). This difference was reflected in the responses observed during the assays of laboratory and clinical enterobacteria where the latter enzymes gave output potential reductions at 30 min above 50 mV while VIM type producers exhibited on average lower signals with high variation (− 29 ± 31 mV; Fig. 4). In strains producing OXA-48, GES type carbapenemases or non-carbapenemases a voltage fall that was lower than that of the combined controls was evident (Fig. 4). The high variation observed in the ISFET responses when VIM producers were assayed was most probably due to different levels of the enzyme in the periplasm of the respective strains. In species such as *E. cloacae* and *P. mirabilis* it seems that VIM production is too low to be detected in 100% of the cases, probably due to low copy number of the gene (likely caused by plasmid stability issues or chromosome incorporation) or inefficient localization in the periplasm.

With the experimental setup used in this study sensitivity is considerably lower for VIM and OXA-48 carbapenemases compared to the pH indicator techniques. Yet, the results obtained permitted a quantitative assessment of the phenomena taking place during application of the latter methodologies while they could serve as a proof of concept for an investment in the development of more sensitive carbapenemase detecting ISFETs. Sensitivity could be improved either by introducing modifications that would increase the concentration of the enzymes in solution (e.g. reaction volume reduction or use of cell wall disruption agents with no buffering effects) or through signal amplification. A considerable volume reduction even below 10 μl could be achieved through the use of a REFET instead of a conventional reference electrode while signal amplification may be enabled either electronically or by coupling the ISFET with an actuator electrode of a coulometric sensor that would permit titration of the acidic hydrolysis product^23,29^. The successful implementation and commercialization of the ISFET technology in microchips used for the detection of the protons produced during DNA polymerization reactions (e.g. IonTorrent, DNA Electronics-Genalysis;^28,30^) suggest that similar setups may be used for the massive, parallel and sensitive detection of imipenemase activity in bacteria producing even unknown carbapenemases directly in clinical samples.

## Materials and methods

### Laboratory strains and bla-carrying plasmids

Natural plasmids and recombinant vectors expressing various β-lactamases were hosted by the following *E. coli* laboratory strains: *E. coli* DH5α [F^–^, *endA1*, *glnV44*, *thi-1*, *recA1*, *relA1 gyrA96*, *deoR*, *nupG*, *purB20*, φ80d*lacZ*ΔM15, Δ(*lacZYA-argF*)U169, hsdR17(*r*_*K*_^–^*m*_*K*_^+^), λ^–^), *E. coli* C600Z1 (*sp*^*r*^*, laci*^*q*^*, PN25-tetR, lacY1, leuB6, mcrB* + *, supE44, thi-1, thr-1, tonA21*), *E. coli* JM109 [*endA1, glnV44, thi-1, relA1 gyrA96, recA1, mcrB*^+^*,* Δ*(lac-proAB), e14-, [*F' *traD36 proAB*^+^ *lacI*^*q*^* lacZΔM15],* hsdR17(*r*_*K*_^*-*^*m*_*K*_^+^)] and *E. coli* 1R716 (*lac*-, *rif*^*r*^). High level expression of NMC-A, VIM-27, CMY-2, CTX-M-15 and, GES-5 was achieved using the recombinant plasmids pNTN3-*bla*_NMC-A_ (*E. coli* JM109), pBC-*bla*_VIM-27_, pBC-*bla*_KPC-2_, pBC-*bla*_CMY-2_, pBC-*bla*_CTX-M-15_ and, pBC-*bla*_GES-5_ (*E. coli* DH5α)^31–36^. In the latter clones, the bla genes were expressed under the control of the respective natural promoters. The open reading frames of VIM-1, NDM-1, IMP-1 and, OXA-48 were cloned into the BamHI and KpnI sites of the pZE21-MCS vector and the resulting plasmids (pZ-*bla*) were used to transform the TetR producing *E. coli* C600Z1 strain^37^. Integrity of the cloned regions and of the expression module was confirmed by Sanger sequencing using the primers pZE21-Fs (5′-TGGCAATTCCGACGTCTAAG-3′) and pZE21-Rs (5′-GTCTAGGGCGGCGGATTTG-3′). In these clones expression of β-lactamases was controlled by the pTetOL1 promoter/operator and was inducible by tetracycline analogues^38^.

### Clinical isolates

A total of 83 non-repetitive enterobacterial clinical strains isolated in Greek hospitals were analyzed. The collection comprised of 35 *Klebsiella pneumoniae* isolates producing at least one carbapenemase type (10 VIM, 5 NDM, 1 IMP, 7 KPC, 5 OXA-48, 1 GES-6, 3 co-producing VIM and KPC and 3 co-producing VIM and NDM β-lactamases) and 12 isolates producing various types of serine reactive β-lactamases not hydrolyzing carbapenems. Additionally 18 *E. coli* isolates were assayed from which eight were producing a carbapenemase (two VIM, five KPC and one GES-5) and 10 expressing for β-lactamases with no such functionality. The collection included also 10 VIM producing enterobacterial isolates belonging to *Enterobacter cloacae* (n = 2), *Proteus mirabilis* (n = 5), *Providencia stuartii* (n = 2) and *Serratia liquefaciens* (n = 1) as well as carbapenemase negative *E. cloacae* (n = 3), *Klebsiella aerogenes* (n = 4) and *P. mirabilis* (n = 1). Species identification, antimicrobial susceptibility and β-lactamase content characterization were performed as described previously^7,39^. The genetic diversity of the isolates expressing the same β-lactamases has been previously asserted using Multi Locus Sequence Typing (MLST) and Restriction Fragment Length Polymorphism/Pulse Field Gel Electrophoresis (RFLP-PFGE).

### Carbapenem MIC measurements

Imipenem and meropenem MICs were determined using the broth micro-dilution in Mueller–Hinton in accordance with EUCAST guidelines. During the testing of the strains carrying the pZE21 recombinant plasmids, anhydrotetracycline was included in the broth at a concentration of 200 ng/ml, for induction of the cloned β-lactamases.

### Preparation of crude protein extracts

β-Lactamase preparations from the recombinant *E. coli* clones as well as from the *P. mirabilis* EUG91 strain and its transformant *E. coli* trfEUG91 were obtained after ultra-sonication of cells grown in Luria–Bertani (LB) broth as described previously^39^. Cultures of the pZE21 strains were allowed to reach the late logarithmic phase (OD_600nm_ ≈ 0.5) and then β-lactamase production was induced for 18 h by addition of 200 ng/ml anhydrotetracycline. MβL producers were grown in the presence of 300 μM ZnSO_4_ and β-lactamases were released in 0.05 Μ HEPES buffer pH 7. Sonication of the remaining clones was performed in 0.1 M potassium phosphate buffer pH 7. Total protein content of the extracts was determined by the Bradford method. Imipenem hydrolysis was monitored using UV spectrophotometry at 300 nm at a substrate concentration of 80 μM and pH 7. Carbapenemase concentrations in crude extracts of the recombinant clones were estimated using the measured initial velocities and the published steady state kinetic constants (*k*_cat_ and *K*_m_) for imipenem hydrolysis through the *Michaelis–Menten* equation^39–46^.

### Equipment and application of the ISFET method

The ISFET sensor used in the study is commercially available from WINSENSE Ltd., Thailand (Fig. 1a) and consisted of three terminals (source, drain and a reference electrode substituting the metal gate). Silicon nitride (Si_3_N_4_) made up the gate insulator and the reference electrode was of the Ag/AgCl type (Fig. 1b). A circuit provided by WINSENSE Ltd. was used to keep a stable drain current (I_ds_) for the ISFET operation (Fig. 1b). Output voltage was monitored using a VA18B digital multimeter (Mastech, China) connected to a PC. The ISFET was placed into the bottom of a custom made flow cell provided by Winsense Ltd. with the reference electrode adapted in the top of the compartment that had a volume capacity of approximately 0.5 ml (Fig. 1a). Solutions were applied to the apparatus using a 5 ml syringe through a T tubing connector (Bio-Rad, USA). The output voltage was linearly depended on the pH of the solution (one pH unit fall corresponded to approximately 45 mV of voltage reduction). Performance of the device was routinely assessed using standard buffers of pH values 4, 7 and 10 (ThermoFisher Scientific, USA).

The ability of the sensor to detect carbapenem hydrolysis (Fig. 1b) was initially assessed using β-lactamase preparations obtained from the recombinant *E. coli* clones. For this, 10 mg/ml imipenem stock solutions of the commercially available imipenem/cilastatin formulation (50 mg imipenem/50 mg cilastatin) were prepared in water, 10 mM Tris/HCl pH 7 or 0.3 mM ZnSO_4_ with pH adjusted at 7.2 using drops of 10 N NaOH. Each experiment was performed by diluting a quantity of the enzyme in 0.5 ml of solution which was then mixed with 1 ml of the imipenem stock yielding a final substrate concentration of 6.67 mg/ml. The total 1.5 ml of the reaction was aspired with a 5 ml syringe and transferred to the ISFET flow cell. Voltage was monitored continuously for 20–30 min and the resulting curve was compared with control measurements of imipenem. The flow cell was washed with 20 ml of deionized water between each measurement. Preparations containing VIM-1, NDM-1 and IMP-1 metallo-β-lactamases, KPC-2, NMC-A and OXA-48 serine reactive carbapenemases as well as CMY-2 and CTX-M-15 β-lactamases-not exhibiting meaningful imipenem hydrolysis-were assayed. Significant voltage reduction was observed only for the efficient enzymes indicating that the method is able to specifically detect the presence of a carbapenemase in solution. The ZnSO_4_ solution yielded the best results and it was used in subsequent experiments. In order to estimate the lowest detection limits for each carbapenemase various quantities of protein extracts were assayed and the rate of voltage reduction was measured for the stable phase of the control corrected 30 min curves. A voltage reduction of 10 mV in 30 min (rate ≈ − 0.33 mV∙min^−1^) was set as the hydrolysis detection threshold (discussed in the last paragraph of the section). Detection limits were determined through mV∙min^−1^ versus [E] plots and were expressed as the enzyme concentration contained in the reaction in nM resulting in a voltage reduction rate of − 0.33 mV∙min^−1^. Imipenem hydrolysis rates by the carbapenemase preparations were also measured through UV spectrophotometry at 300 nm in parallel experiments using 80 μΜ substrate in the ISFET measurement solution.

Whole cell assays were performed using the same setup as above. Bacteria were grown in Tryptic Soy Agar (TSA) containing 300 μM ZnSO_4_ at 37 °C for 16 h. For the pZE21 clones 200 ng/ml of anhydrotetracycline was added in the medium for induction of the cloned β-lactamase genes. Heavy bacterial suspensions were prepared in 0.5 ml of the ZnSO_4_ solution (5 full 10 μl loops for all but the pZ-NDM-1 and pE-NMC-A clones for which suspensions having a density of 1 and 2 in the MacFarland scale were used due to rapid imipenem hydrolysis when using denser preparations). 1 ml of the imipenem stock solution was added and the mixture was thoroughly vortexed and transferred into the ISFET apparatus using a 5 ml syringe. The output voltage was monitored for 30 min. Control measurements of bacterial suspensions without imipenem and of the imipenem solution were used to correct the bacterium/imipenem measurement through subtraction of normalized curves according to the following equations:

5 $$\Delta {\text{V}}_{{{\text{rel}}}} \left( {\text{t}} \right) \, = {\text{ V}}\left( {\text{t}} \right) \, - {\text{ V}}_{{{\text{max}}}}$$

6 $$\Delta {\text{V}}_{{{\text{cor}}}} \left( {\text{t}} \right)^{{{\text{bacterium}}/{\text{imipenem}}}} = \Delta {\text{V}}_{{{\text{rel}}}} \left( {\text{t}} \right)^{{{\text{bacterium}}/{\text{imipenem}}}} {-} \, (\Delta {\text{V}}_{{{\text{rel}}}} \left( {\text{t}} \right)^{{{\text{bacterium}}}} + \Delta {\text{V}}_{{{\text{rel}}}} \left( {\text{t}} \right)^{{{\text{imipenem}}}} )$$

where ΔV_rel_(t) is the relative voltage change at each time point that was obtained by subtracting the maximum voltage reading observed during the course of each measurement (V_max_) from the voltage reading at each time point (V(t)) and ΔV_cor_(t)^bacterium/imipenem^ is the corrected relative voltage change of the bacterium/imipenem mix.

Between each measurement the flow cell was washed with 50 ml deionized water, 20 ml of 70% v/v ethanol and again with 50 ml of deionized water and finally it was equilibrated with the ZnSO_4_ solution.

After experiments, the ISFET was washed as above and dried while the reference electrode was stored in a saturated KCl solution (Orion electrode storage solution, ThermoFisher Scientific).

The corrected voltage drop at 30 min that would yield the optimal results in terms of sensitivity and specificity when the method is applied in whole cell assays was estimated using the Receiver Operating Characteristic (ROC) curve analysis^20^ in Prism 6 software (GraphPad, USA). The signals obtained from all measurements of carbapenemase negative strains were grouped in the “Control” column while those of carbapenemase producers in the “Patient” column and the data were analyzed using the software’s default parameters. A signal of < − 9.5 mV at 30 min yielded a sensitivity of 80.82% [95% Confidence Interval (CI): 69.92–89.10%] and 100% specificity (95% CI 91.96–100%). Hence the detection threshold was set at a voltage drop of − 10 mV at 30 min.

## Comparisons with a colorimetric technique

Clinical strains which yielded false negative results with the ISFET method were also assayed with the commercial pH indicator colorimetric technique RAPIDEC CARBA NP (BioMérieux, France). Strains found true positive and true negative, regarding carbapenemase production, with the ISFET method were also tested for control purposes. Bacteria were grown on TSA containing 300 μM ZnSO_4_ at 37 °C for 16 h and the test was performed and interpreted according to the manufacturer’s instructions.
